# The Leber Congenital Amaurosis Protein AIPL1 and EB Proteins Co-Localize at the Photoreceptor Cilium

**DOI:** 10.1371/journal.pone.0121440

**Published:** 2015-03-23

**Authors:** Juan Hidalgo-de-Quintana, Nele Schwarz, Ingrid P. Meschede, Gabriele Stern-Schneider, Michael B. Powner, Ewan E. Morrison, Clare E. Futter, Uwe Wolfrum, Michael E. Cheetham, Jacqueline van der Spuy

**Affiliations:** 1 Department of Ocular Biology and Therapeutics, UCL Institute of Ophthalmology, London, United Kingdom; 2 Department of Cell Biology, UCL Institute of Ophthalmology, London, United Kingdom; 3 Cell and Matrix Biology, Institute of Zoology, Johannes Gutenberg University of Mainz, Mainz, Germany; 4 Section of Ophthalmology and Neuroscience, Leeds Institute of Molecular Medicine, St James’s University Hospital, Leeds, United Kingdom; National Eye Institute, UNITED STATES

## Abstract

**Purpose:**

The aim of this study was to investigate the interaction and co-localization of novel interacting proteins with the Leber congenital amaurosis (LCA) associated protein aryl hydrocarbon receptor interacting protein-like 1 (AIPL1).

**Methods:**

The CytoTrapXR yeast two-hybrid system was used to screen a bovine retinal cDNA library. A novel interaction between AIPL1 and members of the family of EB proteins was confirmed by directed yeast two-hybrid analysis and co-immunoprecipitation assays. The localization of AIPL1 and the EB proteins in cultured cells and in retinal cryosections was examined by immunofluorescence microscopy and cryo-immunogold electron microscopy.

**Results:**

Yeast two-hybrid (Y2H) analysis identified the interaction between AIPL1 and the EB proteins, EB1 and EB3. EB1 and EB3 were specifically co-immunoprecipitated with AIPL1 from SK-N-SH neuroblastoma cells. In directed 1:1 Y2H analysis, the interaction of EB1 with AIPL1 harbouring the LCA-causing mutations A197P, C239R and W278X was severely compromised. Immunofluorescent confocal microscopy revealed that AIPL1 did not co-localize with endogenous EB1 at the tips of microtubules, endogenous EB1 at the microtubule organising centre following disruption of the microtubule network, or with endogenous β-tubulin. Moreover, AIPL1 did not localize to primary cilia in ARPE-19 cells, whereas EB1 co-localized with the centrosomal marker pericentrin at the base of primary cilia. However, both AIPL1 and the EB proteins, EB1 and EB3, co-localized with centrin-3 in the connecting cilium of photoreceptor cells. Cryo-immunogold electron microscopy confirmed the co-localization of AIPL1 and EB1 in the connecting cilia in human retinal photoreceptors.

**Conclusions:**

AIPL1 and the EB proteins, EB1 and EB3, localize at the connecting cilia of retinal photoreceptor cells, but do not co-localize in the cellular microtubule network or in primary cilia in non-retinal cells. These findings suggest that AIPL1 function in these cells is not related to the role of EB proteins in microtubule dynamics or primary ciliogenesis, but that their association may be related to a specific role in the specialized cilia apparatus of retinal photoreceptors.

## Introduction

Mutations in the *AIPL1* gene cause the devastating disease Leber’s congenital amaurosis (LCA) [1], which is characterized by profound visual impairment or loss at birth. LCA is a genetically heterogeneous disorder that is typically inherited in an autosomal recessive manner, and has been linked to more than 19 genes involved in the retinoid cycle and phototransduction, photoreceptor transcriptional and translational regulation, photoreceptor morphogenesis, and protein trafficking involving the photoreceptor connecting cilium (Retinal Information Network (RetNet) (https://sph.uth.edu/retnet) and online Mendelian Inheritance in Man (OMIM) 204000).

The underlying mechanism of disease pathogenesis caused by *AIPL1* mutations and the normal function of AIPL1 in photoreceptor cells has not been fully elucidated, but it appears to function as a photoreceptor-specific molecular chaperone. Human AIPL1 is 42% identical to the human AIP (aryl hydrocarbon receptor (AHR) interacting protein) and similar to FKBP51 and FKBP52, all of which are members of a group of co-chaperones that interact specifically with the molecular chaperone Hsp90 via a conserved tetratricopeptide repeat (TPR) domain [2,3]. The FKBP51 and FKBP52 co-chaperones have been widely studied with respect to their role in the transcriptional control of Hsp90-bound transcription factors, including members of the hormone-dependent superfamily of nuclear receptors. FKBP52 potentiates transcriptional activity via a dual mechanism involving the increased ligand-binding affinity of the Hsp90-associated receptor, and by targeting the efficient microtubule-dependent retrotranslocation of the signalling complex mediated by the direct interaction of FKBP52 with the dynein molecular motor [4–8]. Moreover, this mechanism is facilitated by the direct interaction of FKBP52 with tubulin thereby linking the heterocomplex to cytoskeletal tracts, and an FKBP52 microtubule depolymerization activity has revealed a role in microtubule dynamics [9–11]. In contrast, FKBP51, which is unable to bind dynein, has been shown to negatively regulate transcriptional activity by reducing the ligand binding affinity and efficiency of nuclear translocation [12]. When the nuclear translocation rate is impaired, the Hsp90-bound receptors became highly sensitive to proteasomal degradation [13]. AIP, similar to FKBP51 and FKBP52, modulates the transcriptional activity of the Hsp90-bound aryl hydrocarbon receptor [14].

An analogous role for AIPL1 in transcriptional control has not been demonstrated. However, the ablation or hypomorphic expression of *AIPL1* in transgenic mice revealed a role for AIPL1 in cyclic nucleotide signalling [15, 16]. The loss or reduction of *AIPL1* expression in mice leads to the post-transcriptional loss of all three subunits of cGMP phosphodiesterase (PDE), a critical component of the phototransduction cascade required for normal vision. Specifically, AIPL1 is required for the stability of the catalytic PDEα subunit, the loss of which results in the misassembly of the PDE holoenzyme and the rapid proteasomal degradation of all three PDE subunits [17]. We have shown that AIPL1 is critical for proteostasis in photoreceptor cells, not only through its interaction with the Hsp90 chaperone machinery, but also through its interaction with NUB1, a protein that directly binds the proteasome to target the degradation of substrate proteins [3,18,19]. Indeed, a recent report identified PDE as an Hsp90 substrate in retina, suggesting that AIPL1 together with Hsp90 function in a chaperone heterocomplex that is essential for PDE biogenesis and maturation [20]. However, the precise molecular mechanisms of how AIPL1 maintains the stability of PDEα in the biosynthetic pathway and assembly of the PDE holoenzyme is unknown. While the PDE holoenzyme is functional in the photoreceptor outer segments, AIPL1 is compartmentalized predominantly in the remainder of the photoreceptors from the synapse to the inner segment, with an enrichment of AIPL1 detected in the region of the photoreceptor connecting cilium [21, 22]. It is unknown whether the interaction of AIPL1 with PDE is coordinated with the order of events leading to PDE protein translocation to the outer segment via the connecting cilium following protein synthesis in the inner segment.

In this study, we identified a novel interaction between AIPL1 and the microtubule end-binding (EB) proteins, EB1 and EB3. The EB proteins are microtubule plus-end tracking proteins (+TIPs) that exchange rapidly at growing microtubule ends and have an important function in microtubule dynamics [23–29]. The three conserved members of the EB family (EB1, 2 and 3) are characterised by an N-terminal microtubule-binding calponin homology domain, followed by a coiled coil dimerization domain that overlaps with a unique EB homology domain, and a C-terminal negatively charged amino acid tail [30–32]. The EB proteins exist as homodimers, although EB1 and EB3 also form a heterodimeric complex and exhibit a significant degree of functional redundancy [33]. The EB proteins interact with numerous +TIP proteins, including the adenomatous polyposis coli (APC) tumor suppressor protein, the microtubule-actin crosslinking factor (MACF), the mitotic centromere-associated kinesin (MCAK), the cytoplasmic linker protein of 170 kDa (CLIP-170) and the large dynactin subunit p150Glued [23,26]. These associations are important in regulating the reciprocal interaction of microtubules with various cellular structures, including the cell cortex and membranes, the mitotic kinetochore and different cellular organelles. EB complexes at the centrosome are thought to contribute to centrosomal microtubule organization and to stabilize microtubule outgrowth through minus end anchoring. Indeed, EB1 and EB3 have been localized to primary cilia in mammalian cells where they are required for cilia formation and assembly through minus end anchoring at the basal body, thereby facilitating vesicular trafficking to the cilium base [34,35].

Here, we examined the association of AIPL1 and the EB proteins with the cellular microtubule network, primary cilia in cultured cells and cilia of retinal photoreceptor cells in order to gain further insight into the function of AIPL1.

## Methods

### Yeast two-hybrid analysis

The CytoTrapXR yeast two-hybrid screen (Stratagene, La Jolla, CA) was performed as described previously [3]. In brief, the *Saccharomyces cerevisiae* strain cdc25Hα was co-transformed with pSos-AIPL1 (full-length wild type human AIPL1) [3] and a bovine retinal cDNA library fused to a myristoylation sequence in pMyr using the lithium acetate method [36]. The cdc25Hα strain harbours a temperature sensitive mutation in the cdc25 gene such that growth at the restrictive temperature (37°C) can only occur following the recruitment of the pSos bait to the membrane by interaction with the membrane-targeted pMyr prey, and subsequent complementation of the mutant cdc25 gene by the human Sos homologue in the activation of the Ras pathway necessary for cell growth and survival. Putative positive colonies that grew at 37°C on synthetic media containing galactose (gal) but lacking leucine (-L) and uracil (-U) were subsequently excluded from further analysis if they were still able to grow at 37°C on gal(-U) once cured of pSos-AIPL1. The pMyr-cDNA plasmids were isolated from the positive colonies and sequenced using an ABI Sequencer and Big Dye Terminator (PerkinElmer Life Sciences, Wellesley, MA). In order to confirm the interactions, pSos-AIPL1 and the pMyr-cDNA were retransformed in cdc25Hα. CytoTrapXR pSos and pMyr positive and negative interaction controls were used throughout. The pSos-AIPL1 mutants H82Y, A197P, C239R, G262S, W278X and R302L have been described previously [3].

### Cell culture

Human SK-N-SH neuroblastoma cells and ARPE-19 retinal pigment epithelial cells were cultured in Dulbecco’s modified Eagle’s medium (DMEM)/F12 (Invitrogen, Paisley, UK) supplemented with 10% (v/v) fetal bovine serum (FBS) and penicillin (10,000 U/ml)/streptomycin (10,000 μg/ml). ARPE-19 cells were serum starved (48 h) to induce ciliogenesis [37]. Cells were obtained from the American Type Culture Collection (ATCC, Manassas, VA).

### Immunoprecipitation

SK-N-SH cells were seeded in 6-well plates (Nunc, Fisher Scientific, UK) at a density of 5 X 10^5^ cells per well. Twenty-four hours later, Lipofectamine and Lipofectamine Plus reagent (Invitrogen) were used according to the manufacturer’s instructions to transfect cells with pCMV-Tag3C-AIPL1, pJMA2eGFP-EB1 or pJMA2eGFP-EB3 (400 ng) as indicated with the addition of empty vector as necessary to equalise the total amount of plasmid transfected (800 ng). pCMV-Tag3C-AIPL1 has been described previously [18]. pJMA2eGFP-EB1, pJMA2eGFP-EB2 and pJMA2eGFP-EB3 were constructed in the laboratory of Ewan E. Morrison. 24 h later, cells were lysed in lysis buffer (1% (w/v) *n*-dodecyl β-D-maltoside, 5 mM EDTA, pH 8.0, and 1% (v/v/) protease inhibitor cocktail (Sigma-Aldrich, Dorset, UK) in PBS) at 4°C for 15 min. Myc-AIPL1 was immunoprecipitated from lysates using anti-c-myc 9E10 mouse ascites fluid (Sigma-Aldrich) as described [3]. Co-immunoprecipitation was also performed with non-specific mouse IgG. Immunoprecipitation of myc-AIPL1 and co-immunoprecipitation of EB1-GFP, EB2-GFP or EB3-GFP was detected by western blotting using rabbit anti-AIPL1 [21] and anti-GFP A.v. peptide antibody (BD Biosciences, Oxford, UK) respectively. The rabbit anti-AIPL1 antibody is specific for AIPL1 in human retina [21].

### Immunocytochemistry

SK-N-SH cells or ARPE-19 cells were seeded in 8-well permanox chamber slides (VWR, Lutterworth, UK) at a density of 5 X 10^4^ cells per well. Twenty-four hours later, cells were transfected with pBK-CMV-AIPL1 [18] (100 ng) using Lipofectamine and Lipofectamine Plus reagent (Invitrogen) according to the manufacturer’s instructions. SK-N-SH cells were incubated in complete medium (DMEM/F12 plus 10% (v/v) FBS) in the absence of antibiotics for 24 h following transfection. Cells were treated with nocodazole (2.5 μg/ml) for 1 hour, allowed to recover in complete medium for 5 min or 15 min, and fixed in 100% ice-cold methanol at -20°C for 10 min. ARPE-19 cells were incubated in serum-free medium in the absence of antibiotics for 24 hours following transfection, and then fixed in 4% (v/v) paraformaldehyde for 10 minutes, or 4% (v/v) paraformaldehyde for 10 min followed by 100% ice-cold methanol for 2 min. Following cell fixation, SK-N-SH and ARPE-19 cells were treated in the same way. In brief, cells were washed 3 times in phosphate-buffered saline (PBS), and then incubated in blocking solution (3% (w/v) BSA plus 10% (v/v/) normal donkey or goat serum in PBS) for 30 min at room temperature. Primary antibodies were appropriately diluted in blocking solution and applied to the cells for 1 h at room temperature. Primary antibodies were rabbit anti-AIPL1 (1:250) [21], mouse anti-EB1 (1:250) (BD Biosciences), mouse anti-β-tubulin clone TUB2.1 (1:500) (Sigma-Aldrich), mouse anti-acetylated α-tubulin (1:1000) (Sigma-Aldrich), mouse anti-γ-tubulin GTU-88 (1:1000) (Abcam, Cambridge, UK), rabbit anti-Arl13b (1:500) and rabbit anti-pericentrin (1:1000) (Abcam). Cells were washed 3 times in PBS and incubated with fluorescently labelled secondary antibodies, appropriately diluted in blocking solution, for 45 min at room temperature. Secondary antibodies (1:100) were Cyanine2- and Cyanine3-conjugated donkey anti-mouse, donkey anti-rabbit and goat anti-rat (Stratech, Newmarket, Suffolk, UK). Cells were washed several times in PBS, and incubated with 4’,6-diamidino-2-phenylindole (DAPI) (1:5000) (Sigma-Aldrich) in PBS for 5 minutes to label the nuclei. Cells were mounted in fluorescent mounting media (Dako, Ely, UK). Confocal images were obtained using an LSM700 microscope (Carl Zeiss MicroImaging) and analysed using the LSM Image Browser software (Zen 2009 Light Edition).

### Analysis of co-localization

Co-localization was analysed using the Just Another Co-Localization Plugin (JACoP) [38] for ImageJ (http://rsbweb.nih.gov/ij/). For each co-localization analysis, the Pearson’s intensity correlation (r) and Manders’ overlap coefficient (M1 = fraction of A overlapping B, M2 = fraction of B overlapping A) was measured in the demarcated subcellular area of interest in several cells. Pearson's coefficient is a measure of the portion of variance in one channel that can be correlated with the variance of the other channel and is an indication of the linear dependency between the two colour channels. Pearson’s coefficient scores between (−)1 and (+)1, where (−)1 indicates total exclusion, (+)1 a perfect image registration, and (0) a random localization. All values were normalized to the threshold for Pearson’s coefficient. Manders’ overlap coefficients calculate the contribution of the intensity in each channel that coincides with some intensity in the other channel giving a number between 0 and 1. The unpaired Student’s *t*-test was used to calculate the standard error of the mean (SEM).

### Human Tissue

The use of human tissue was approved by the Eye Tissue Repository Internal Ethics Committee institutional review board (ETR reference: 10/H0106/57-2011ETR16). Informed consent was obtained, and the tenets of the Declaration of Helsinki were adhered to.

### Immunohistochemistry

Immunohistochemistry on unfixed retinal cryosections from adult wild-type C57Bl6 mice and human was performed as described previously [37,39]. Briefly, cryosections were placed on poly-L-lysine-pre-coated coverslips and incubated for 20 min at RT with 0.01% Tween-20 in PBS. After a PBS washing step, sections were incubated with blocking solution (0.5% cold-water fish gelatin plus 0.1% ovalbumin in PBS) and incubated for 2 h followed by overnight incubation with primary antibodies in blocking solution. Primary antibodies were rabbit anti-EB1 (1:500) (E3406, Sigma-Aldrich), anti-EB3 (Santa Cruz), rabbit anti-AIPL1 (1:500) and mouse anti-centrin-3 (1:100). After washing with PBS, sections were incubated with secondary AlexaFluor 488 or AlexaFluor 568 conjugated antibodies (Molecular Probes) and with DAPI (Sigma-Aldrich) for nuclear DNA staining. Control sections for immunospecificity were treated with either primary or secondary antibodies on their own, and the cross-reactivity between the primary and secondary antibodies of each double labelling experiment was tested. Sections were mounted in Mowiol 4.88 (Carl Roth GmbH), and analyzed with a Leica DM-6000B microscope. Images were taken with a DFC 360FX camera (Leica,) and processed with Adobe Photoshop CS.

### Cryo-Immunogold Electron microscopy

Human eyes collected for corneal transplantation from anonymous donors were used. The whole eye globe, after corneal removal, was fixed in 2% paraformaldehyde. Tissue from a 57 year old female fixed 7 hours after death, and from a 66 year old female fixed 8 hours after death, were used. There were no reported ophthalmic problems with either donor. Cryo-immunogold EM was performed on 1 mm square pieces of mid-peripheral temporal neuroretina fixed in 4% paraformaldehyde in PBS, embedded in 12% gelatine and infused with 2.3 M sucrose. 80 nm sections were cut at -120°C and collected in 1:1 2% methylcellulose: 2.3 M sucrose. Sections were labelled with primary antibodies rabbit anti-EB1 (1:50) and anti-AIPL1 (1:50) followed by protein A gold (PAG) as previously described [40]. Following the labelling of sections with anti-EB1 and PAG (10 nm), sections were quenched in 1% glutaraldehyde, blocked and incubated with anti-AIPL1 and PAG (15 nm). The control sections were treated in exactly the same way, but in the absence of anti-AIPL1. Samples were viewed on a JEOL 1010 transmission electron microscope and imaged using Gatan Orius SC1000B charge-coupled device camera.

## Results

### AIPL1 interaction with members of the family of microtubule-associated end-binding (EB) proteins

**Fig 1:**
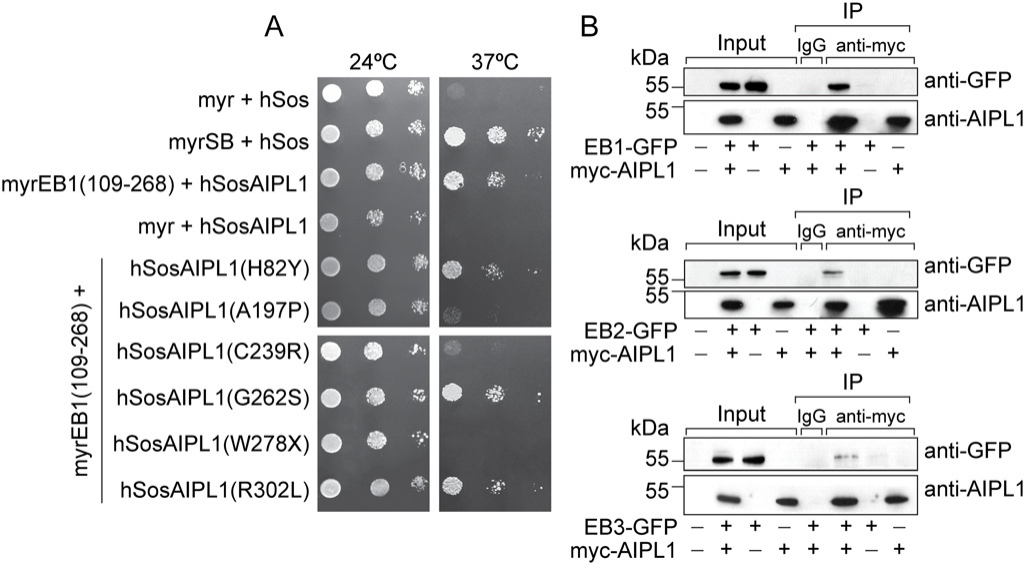
AIPL1 interacts with EB proteins. **A**: Directed yeast two-hybrid interaction of AIPL1 (hSosAIPL1) with EB1 (myrEB1(109–268)). Cdc25α yeast were co-transformed with plasmids as indicated, and serial dilutions of the co-transformants were grown on selective media at the permissive (24°C) and restrictive (37°C) temperatures. **B**: Co-immunoprecipitation of EB1-GFP (upper panel), EB2-GFP (middle panel) and EB3-GFP (lower panel) with myc-AIPL1 from transfected SK-N-SH cells with anti-c-myc 9E10 or non-specific IgG. Samples were resolved by denaturing SDS-PAGE (10%) and proteins were detected with anti-GFP and anti-AIPL1. Molecular weight markers are in kDa.

A CytoTrapXR Y2H screen of approximately 1 X 10^6^ colonies of a bovine retinal cDNA library revealed a novel interaction between AIPL1 and members of the family of microtubule end-binding (EB) proteins. Three cDNA sequences expressed the C-terminal half (amino acid residues 109–268) of the *Bos taurus* microtubule-associated protein RP/EB family member 1 or end-binding protein 1 (EB1), which shares 97% identity with the human homologue in this region. One cDNA sequence was identified as expressing the C-terminal half of the microtubule-associated protein RP/EB family member 3 or EB3, corresponding to amino acid residues 135–266 in *Bos taurus* and residues 135–281 in *Homo sapiens*, which are identical in this region with the exception of a 15 residue insertion in the human homologue. In addition to EB1 and EB3, known AIPL1 interacting proteins (NUB1, Hsp90 and Hsp70) were identified in this screen. The interaction between AIPL1 and EB1 was verified by directed 1:1 Y2H analysis (Fig. 1A). Growth at the restrictive temperature (37°C) was observed when pSos-AIPL1 was co-transformed with pMyr-EB1(109–268) but not with empty pMyr vector, and the growth rate was similar to that of the positive control (pSos co-transformed with pMyr-SB). Next we tested whether the EB proteins EB1, EB2 and EB3 interact with AIPL1 in mammalian cells. For this we co-transfected SK-N-SH neuroblastoma cells with full-length human myc-tagged AIPL1 (myc-AIPL1) and full-length human GFP-tagged EB1 (EB1-GFP), EB2 (EB2-GFP) or EB3 (EB3-GFP), respectively. AIPL1 is expressed specifically and exclusively in the pineal gland and retinal photoreceptors, thereby necessitating the ectopic expression of AIPL1 in neuroblastoma cells which do not express AIPL1 endogenously. In anti-c-myc immunoprecipitations, EB1-GFP, EB2-GFP and EB3-GFP were specifically recovered with myc-AIPL1 (Fig. 1B) confirming their interaction with AIPL1. The EB proteins were not co-immunoprecipitated with anti-c-myc in the absence of myc-AIPL1 or with non-specific mouse IgG. Further studies were subsequently focused on EB1 and EB3 that were identified in the Y2H screen. We also performed reciprocal co-immunoprecipitations of endogenous AIPL1 and the EB proteins from human retinal extracts. We were unable to detect the interaction of endogenous AIPL1 and endogenous EB proteins in human retinal extracts, as the immunoprecipitation of AIPL1 and the EB proteins with their respective available antibodies was highly inefficient (data not shown). It is also possible that the endogenous association of AIPL1 and the EB proteins is transient and dynamic, precluding their detection by co-immunoprecipitation.

### EB1 binding to AIPL1 is altered by LCA-associated sequence variants

Next we examined whether EB1 binding to AIPL1 is altered by LCA-associated mutations in AIPL1 (Fig. 1A). For this we performed directed 1:1 Y2H analyses of AIPL1 harbouring LCA-associated sequence variants (H82Y, A197P, C239R, G262S, W278X and R301L) and pMyr-EB1(109–268). The pSos-AIPL1 mutants are all expressed at similar levels in cdc25Hα yeast, with the exception of R302L which was more abundantly expressed, and W278X which forms detergent insoluble aggregates in cells [3]. Growth at the restrictive temperature (37°C) was observed when pMyr-EB1(109–268) was co-transformed with pSos-AIPL1(H82Y), pSos-AIPL1(G262S) and pSos-AIPL1(R302L), however the interaction and growth was severely compromised with pSos-AIPL1(A197P), pSos-AIPL1(C239R) and pSos-AIPL1(W278X).

### AIPL1 localization with EB1 and the microtubule network

**Fig 2:**
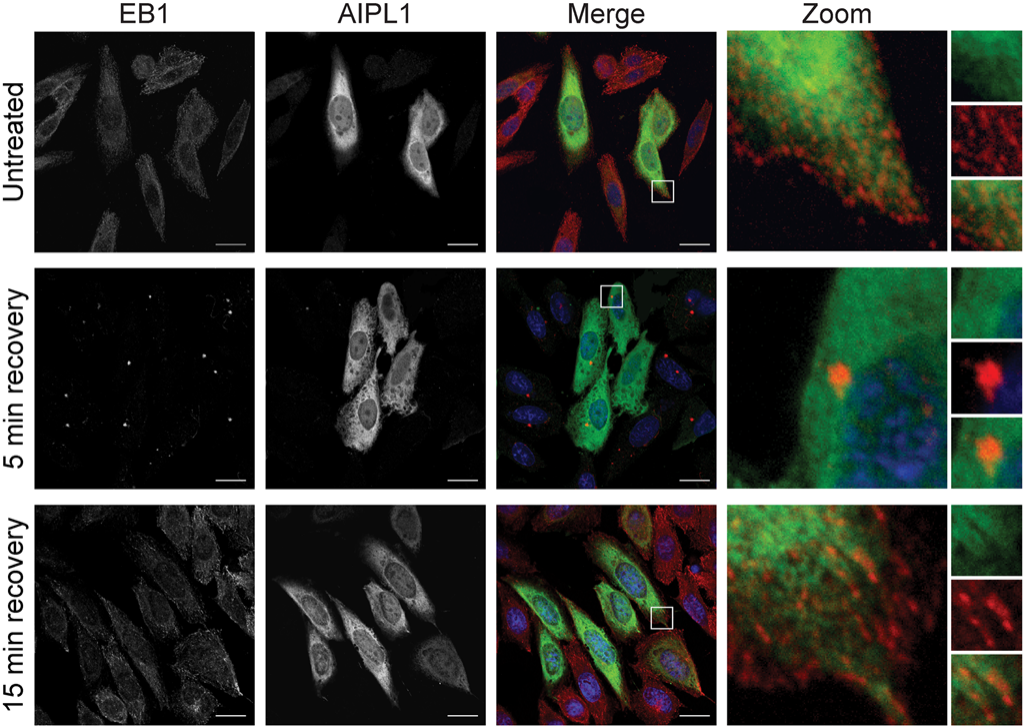
AIPL1 localization overlaps with EB1 at the tips of microtubules: Immunofluorescent localization of AIPL1 (green) and EB1 (red) in SK-N-SH cells in the absence of nocodazole treatment (untreated), and following a recovery period of 5 min (5 min recovery) and 15 min (15 min recovery) after the removal of nocodazole. Nuclei are labelled with DAPI (blue). The zoomed images (zoom) are demarcated by the white squares in the merged images (merge). Split channel inserts for AIPL1 (green) and EB1 (red) are shown in the right hand column. Scale bars: 20 μm.

EB1 was examined by immunofluorescence microscopy in SK-N-SH neuroblastoma cells following transfection with human AIPL1 (untagged). The localization of AIPL1 and endogenous EB1 was examined in the absence of nocodazole treatment, and following a recovery period of 5 and 15 min after the disruption of microtubule polymerisation by nocodazole treatment to assess any change in microtubule seeding or dynamics (Fig. 2). In untreated cells, EB1 was localized to comet-like accumulations at the growing microtubule ends, in accordance with its role as a plus-end tracking protein that exchanges rapidly at microtubule tips. AIPL1 was distributed throughout cells but predominantly in the cytoplasm as shown previously [18]. Disruption of the microtubule network did not affect the subcellular distribution of AIPL1, which was similar in untreated cells and following a recovery period of 5 min and 15 min after the removal of nocodazole. In contrast, nocodazole treatment significantly affected the localization of EB1, which was observed at a fluorescent spot which resembles the microtubule organising center (MTOC) 5 min after the removal of nocodazole. After a recovery period of 15 minutes, EB1 was once again observed at the tips of microtubules as comet-shaped accumulations.

The Just Another Co-Localization Plugin (JACoP) for ImageJ [38] was used to assess the Pearson’s intensity correlation and Manders’ overlap coefficient for the co-localization of endogenous EB1 and AIPL1 in the cytoplasm of SK-N-SH cells (Table 1). The results of these analyses indicate that because AIPL1 is ubiquitously dispersed throughout the cytoplasm, and EB1 is localized specifically to the tips of microtubules and the MTOC, a small fraction of AIPL1 overlaps with the localization of EB1 at both the tips of microtubules in the cytoplasm (M1 = 0.13±0.04, untreated; M1 = 0.14±0.06, 15 min recovery) and at the MTOC (M1 = 0.10±0.04, 5 min recovery). Conversely, a greater proportion of EB1 localization overlaps with that of AIPL1 (M2 = 0.50±0.11, untreated; M2 = 0.65±0.05, 5 min recovery; M2 = 0.51±0.05, 15 min recovery). The intensity correlation reveals that AIPL1 and EB1 do not co-localize specifically at the MTOC 5 min after recovery (r = 0.14±0.04), but that a small fraction of AIPL1 may co-localize with EB1 at the tips of microtubules (r = 0.55±0.04, untreated; r = 0.54±0.04, 15 min recovery). However, the positive correlation between AIPL1 intensity and EB1 intensity at the tips of microtubules is weak with a co-variance of ~30%.

**Table 1:** JACoP analysis of AIPL1 and EB1 co-localization in SK-N-SH cells.

	Pearson’s Coefficient (r)	Manders’ Coefficient (M1)	Manders’ Coefficient (M2)
AIPL1 (A) and EB1 (B) (Untreated)	0.55±0.04	0.13±0.04	0.50±0.11
AIPL1 (A) and EB1 (B) (5 min recovery)	0.14±0.04	0.10±0.04	0.65±0.05
AIPL1 (A) and EB1 (B) (15 min recovery)	0.54±0.04	0.14±0.06	0.51±0.05

M1 = fraction A overlapping B, M2 = fraction B overlapping A

**Fig 3:**
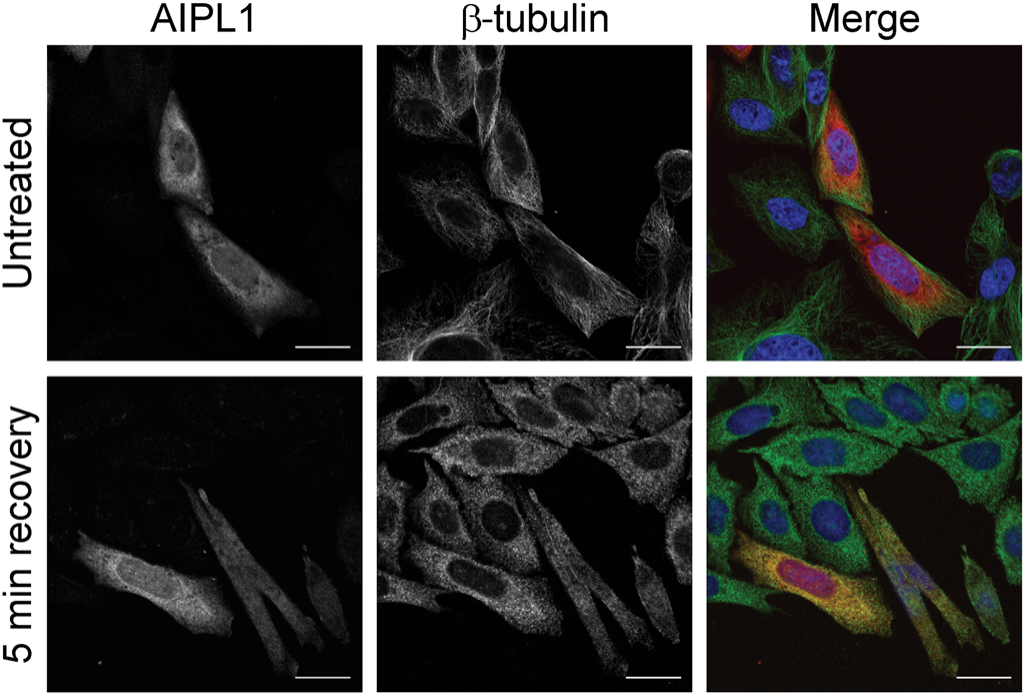
AIPL1 localization overlaps with microtubule-associated β-tubulin: Immunofluorescent localization of AIPL1 (red) and β-tubulin (green) in SK-N-SH cells in the absence of nocodazole treatment (untreated), and following a recovery period of 5 min after the removal of nocodazole (5 min recovery). Nuclei are labelled with DAPI (blue). Scale bars: 20 μm.

EB1 interacts with the large dynactin subunit p150^Glued^ at the ends of growing microtubules [41,42]. Moreover, FKBP52 interacts directly with tubulin and with the dynamitin subunit of dynactin to mediate the retrotranslocation of an FKBP52:Hsp90 associated heterocomplex [4–11]. Therefore, we examined the co-localization of AIPL1 with the microtubule network in untreated and nocodazole treated SK-N-SH cells (Fig. 3).

In untreated cells, a small fraction of AIPL1 overlapped with the β-tubulin subunit of microtubules (M1 = 0.30±0.05), and a larger fraction of the β-tubulin subunit overlapped with AIPL1 (M2 = 0.65±0.04) (Fig. 3, Table 2). The greater M1 value for AIPL1 and the β-tubulin subunit compared to that for AIPL1 and EB1 is a reflection of the fact that whilst microtubules are spread throughout the cytoplasm, EB1 is localized to the tips of microtubules only. The intensity correlation (r = 0.59±0.05) suggests that a small proportion of AIPL1 may co-localize with the β-tubulin subunit of microtubules (co-variance = ~37%). Disruption of microtubule polymerization with nocodazole resulted in dispersion of the β-tubulin subunits throughout the cytoplasm and co-localization with AIPL1 (5 min recovery) (M1 = 0.72±0.07; M2 = 0.80±0.07; r = 0.90±0.01; co-variance = 81%).

**Table 2:** JACoP analysis of co-localization.

	Pearson’s Coefficient (r)	Manders’ Coefficient (M1)	Manders’ Coefficient (M2)
AIPL1 (A) and β-tubulin (B) (Untreated)	0.56±0.05	0.30±0.05	0.59±0.04
AIPL1 (A) and β-tubulin (B) (5 min recovery)	0.90±0.01	0.72±0.07	0.80±0.07
Pericentrin (A) and EB1 (B)	0.79±0.01	0.82±0.06	0.79±0.12
ARL13b (A) and EB1 (B)	0.05±0.03	0.02±0.02	0.02±0.02
γ-tubulin (A) and AIPL1 (B)	-0.08±0.06	0.17±0.14	0.04±0.03
Acetylated α-tubulin (A) and AIPL1 (B)	0.04±0.04	0.05±0.07	0.16±0.10

M1 = fraction A overlapping B, M2 = fraction B overlapping A

### EB1 and AIPL1 localization in primary cilia

**Fig 4:**
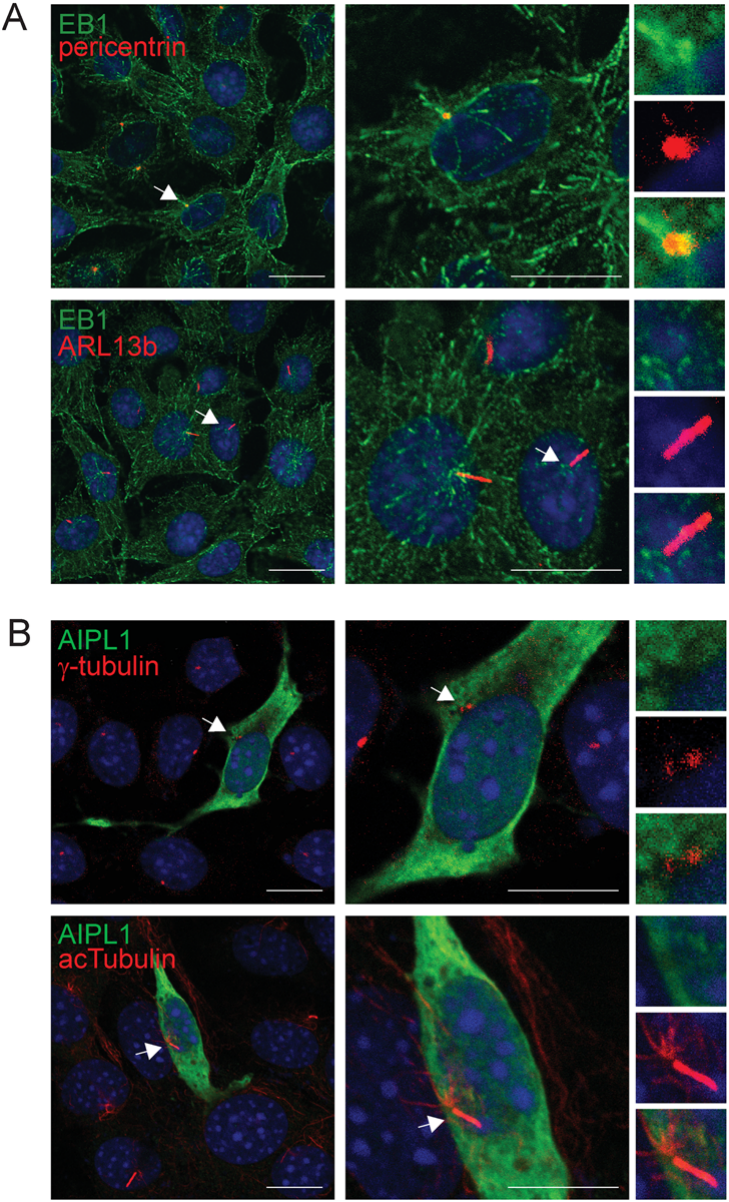
EB1, but not AIPL1, localizes to primary cilia in ARPE-19 cells. **A**: Immunofluorescent localization of EB1 (green) with the cilia markers pericentrin (red) and ARL13b (red) in ARPE-19 cells. Nuclei are labelled with DAPI (blue). The white arrows demarcate the localization of EB1, pericentrin and ARL13b at the primary cilia. **B**: Immunofluorescent localization of AIPL1 (green) with the cilia markers γ-tubulin (red) and acetylated α-tubulin (red) in ARPE-19 cells. Nuclei are labelled with DAPI (blue). The white arrows demarcate the localization of γ-tubulin and acetylated α-tubulin at the primary cilia. The split channel inserts are shown in the right hand column. Scale bars: 20 μm.

EB1 and EB3, but not EB2, localize to the centriole/basal body of primary cilia in cultured cells, and are required for primary cilia biogenesis via a centrosomal mechanism [34,35]. Therefore, the localization of endogenous EB1 and AIPL1 was examined in primary cilia of ARPE-19 cells (Fig. 4). Since ARPE-19 cells do not express endogenous AIPL1 we transfected them with untagged AIPL1. In accordance with the known localization of EB1 at centrioles [33, 34], EB1 co-localized with the centrosomal marker pericentrin in primary cilia (r = 0.79±0.01, M1 = 0.82±0.06, M2 = 0.79±0.12) (Fig. 4A, Table 2). As expected, EB1 did not co-localize with the ciliary membrane marker ARL13b [43] (r = 0.05±0.03, M1 = 0.02±0.02, M2 = 0.02±0.02) (Fig. 4A, Table 2). These findings confirm the specific localization of EB1 to the centrosome near the centrioles in primary cilia but not extending into the primary cilium in accordance with published data.

The localization of AIPL1 to primary cilia in ARPE-19 cells was examined with the basal body marker γ-tubulin and the ciliary marker acetylated α-tubulin (Fig. 4B). AIPL1 was not enriched at the basal body of primary cilia in ARPE-19 cells (Fig. 4B, Table 2). The negative intensity correlation is in accordance with the mutual exclusion of AIPL1 and γ-tubulin at the basal body (r = -0.08±0.06, M1 = 0.17±0.14, M2 = 0.04±0.03). Similarly, AIPL1 did not co-localize with acetylated α-tubulin indicated by the low intensity correlation (r = 0.04±0.04, M1 = 0.05±0.07, M2 = 0.16±0.10). Together, these results reveal that AIPL1 does not localize to primary cilia in ARPE-19 cells. As AIPL1 expression is photoreceptor specific, one possibility is that the cellular machinery necessary for recruitment of AIPL1 to the specialized non-motile photoreceptor sensory cilium, which differs both structurally and functionally from primary cilia, is lacking in ARPE-19 cells. To test this hypothesis we performed high resolution imaging of AIPL1 and EB protein localization in retina.

### AIPL1 and EB1 localization in photoreceptor sensory cilia

In the human retina, AIPL1 is localized from the photoreceptor synapses in the outer plexiform layer to the photoreceptor inner segments where is appears enriched at the connecting cilium [21]. Proteomic analysis of photoreceptor connecting cilia detected both AIPL1 and all three members of the family of EB proteins (EB1, EB2, and EB3) (www.ciliaproteaome.org) [44,45]. Moreover, EB2 has been detected in the connecting cilia of photoreceptor cells in rat retinal cryosections [46]. Therefore, we examined the localization of AIPL1 and the EB proteins, EB1 and EB3, in photoreceptor connecting cilia.

**Fig 5:**
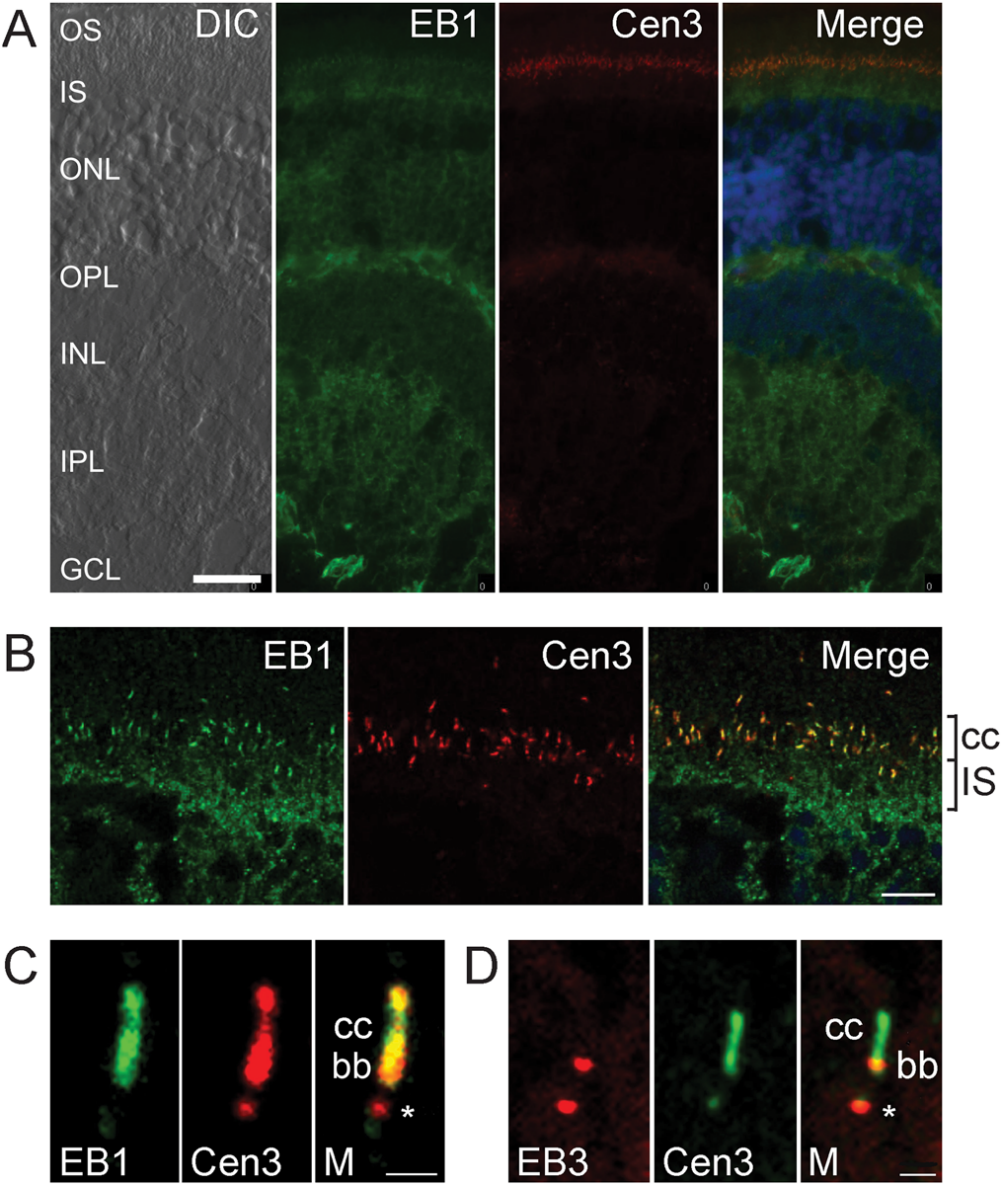
Endogenous EB1 and EB3 co-localize with the ciliary marker centrin-3 in mouse retinal cryosections. **A**: Immunohistochemical localization of EB1 (green) and centrin-3 (red) in mouse retinal cryosections. Nuclei are labelled with DAPI (blue). The DIC (differential interference contrast) image on the left illustrates the laminated retinal cytoarchitecture, labelled as follows: ganglion cell layer (GCL), inner plexiform layer (IPL), inner nuclear layer (INL), outer plexiform layer (OPL), outer nuclear layer (ONL), photoreceptor inner segments (IS) and photoreceptor outer segments (OS). **B**: High magnification of the immunohistochemical localization of EB1 (green) and centrin-3 (red) in photoreceptors. The photoreceptor inner segments (IS) and connecting cilia (cc) are labelled. **C**: High resolution image of EB1 (green) and centrin-3 (red) in the photoreceptor connecting cilium. The connecting cilium (cc) and basal body (bb) are labelled and an asterisk demarcates the cilia-associated centriole. **D**: High resolution image of EB3 (red) and centrin-3 (green) in the photoreceptor connecting cilium. The connecting cilium (cc) and basal body (bb) are labelled, and the cilia-associated centriole is demarcated by an asterisk. Scale bars: A, B: 10 μm; C, D: 1 μm.

In unfixed mouse retina cryosections, EB1 was detected throughout the retina from the inner retina ganglion cell layer to the outer retina photoreceptors (Fig. 5A). High-resolution imaging of the photoreceptor connecting cilium between the photoreceptor inner and outer segments revealed the localization of the ciliary marker centrin-3 (red in Fig. 5C, green in Fig. 5D) [47] to the cilia-associated centrioles (demarcated by an asterisk), basal body (bb) at the proximal end of the connecting cilium, and connecting cilium (cc) extending from the basal body. EB1 (green) co-localized with centrin-3 (red) in the basal body (bb) and connecting cilium (cc) (Fig. 5B, C). In comparison, EB3 (red) co-localized with centrin-3 (green) in the cilia-associated centrioles (asterisk) and basal body (bb) at the proximal end of the connecting cilium (Fig. 5D). These findings reveal the association of the EB1 and EB3 proteins with the specialized ciliary apparatus of photoreceptors.

**Fig 6:**
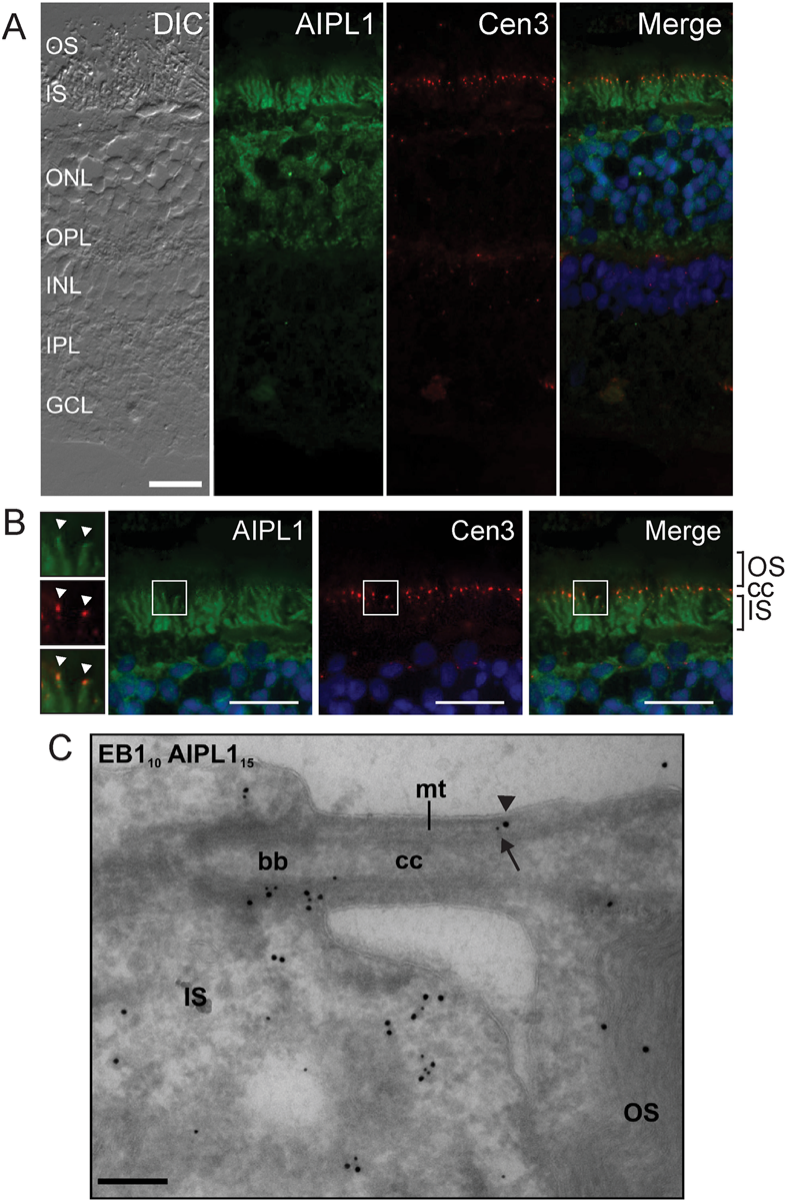
Endogenous AIPL1 co-localizes with ciliary markers and EB1 in human retinal cryosections. **A**: Immunohistochemical localization of AIPL1 (green) and centrin-3 (red) in unfixed human retinal cryosections. Nuclei are labelled with DAPI (blue). The labelling on the DIC (differential interference contrast) image is: ganglion cell layer (GCL); inner plexiform layer (IPL); inner nuclear layer (INL); outer plexiform layer (OPL); outer nuclear layer (ONL); photoreceptor inner segments (IS) and photoreceptor outer segments (OS). **B**: Zoomed image of the immunohistochemical localization of AIPL1 (green) and centrin-3 (red) in photoreceptor cells. The white squares demarcate a further zoomed area (left hand column) highlighting the enrichment of AIPL1 (green) and centrin-3 in the cilia (white arrowheads). Nuclei are labelled with DAPI (blue). Inner segments (IS); connecting cilia (cc); outer segments (OS). Scale bar: 10 μm. **C**: Immunogold labelling of EB1 and AIPL1 in human retinal cryosections. 80 nm human retinal cryosections were double-labelled with antibodies against EB1 (PAG 10 nm, small arrow) and AIPL1 (PAG 15 nm, large arrowhead). Inner segments (IS); outer segments (OS); connecting cilium (cc); basal body (bb); microtubules (mt). Scale bar: 200 nm.

The use of a human-specific AIPL1 antibody necessitated the use of human donor retinas in examining the localization of AIPL1 in photoreceptor connecting cilia. In unfixed human retina cryosections, the localization of AIPL1 extended from the photoreceptor synapses in the outer plexiform layer to the photoreceptor inner segments as previously described [21], and AIPL1 co-localized with centrin-3 in the connecting cilium (Fig. 6A, B). The co-localization of AIPL1 and EB1 in fixed human retina was also assessed by cryo-immunogold electron microscopy in order to gain further insight into their co-localization at the ultrastructural level (Fig. 6C). Co-localization of EB1 (labelled with 10 nm protein A gold) and AIPL1 (labelled with 15 nm protein A gold) was detected in the connecting cilium and around the basal region of the inner segments. Within the connecting cilium, both EB1 and AIPL1 were co-localized in the region of the basal body and the microtubule-based axoneme. In control sections, 15 nm protein A gold particles were not detected with the EB1 (10 nm protein A gold) in the absence of anti-AIPL1 (data not shown), confirming the specific co-localization of AIPL1 with EB1.

## Discussion

In this study, a novel interaction between AIPL1 and members of the family of end-binding proteins, EB1 and EB3, was identified. AIPL1 was found to interact with the C-terminal domain of EB1 and EB3, encompassing the EB homology domain and C-terminal tail. Numerous microtubule plus-end interacting proteins interact with this domain through one of two mechanisms. The first involves the specific recognition of a microtubule tip localization signal, an SxIP motif embedded within a disordered sequence, by the EB homology domain, as exemplified by the interaction with the APC tumor suppressor protein [48]. The second mechanism involves the interaction of the evolutionary conserved EEY/F motif at the extreme C-terminus of the EB proteins with the cytoskeleton associated protein glycine-rich (CAP-Gly) domain of +TIP proteins, such as the large dynactin subunit p150^Glued^ and the cytoplasmic linker protein of 170 kDa (CLIP-170) [48]. The SxIP motif and CAP-Gly domain are not conserved in AIPL1, suggesting that the interaction of AIPL1 with the EB proteins is mediated via a different mechanism.

The interaction of EB1 with the AIPL1 variants H82Y, G262S and R301L was unaffected. Molecular genetic diagnosis of AIPL1 patients coupled with *in silico* analysis and estimates of pathogenic probability of these missense variants suggest that they may be of uncertain pathogenic status or benign rare variants [49,50]. In contrast, the interaction of EB1 with the aggregation-prone AIPL1 mutant W278X and with the AIPL1 TPR mutants A197P and C239R was severely compromised. These are confirmed disease-causing mutations in AIPL1. Therefore, the interaction of AIPL1 with EB proteins might reflect an important function of AIPL1 that is compromised in LCA. It is also possible, however, that the compromised interaction of W278X, A197P and C239R with EB1 is a consequence of the misfolding of these AIPL1 mutants.

We examined the localization of the EB proteins and AIPL1 in cells in order to gain further insight into their interaction. Manders’ overlap coefficient revealed that a very small proportion of AIPL1 is coincident with EB1 at the tips of microtubules, but the weak intensity correlation suggests a largely non-specific overlap at this localization. Moreover, ectopically expressed untagged AIPL1 did not co-localize with endogenous EB1 at the microtubule organising centre. Similarly, the relatively weak intensity correlation of AIPL1 with the β-tubulin subunit of the microtubule network supports a largely non-specific overlap due to the dispersed localization of AIPL1 throughout the cytosol. Whilst the localization of both EB1 and β-tubulin was significantly disrupted by treatment with nocodazole, the distribution of AIPL1 remained unchanged. These data suggest that the interaction of AIPL1 with EB1 may not be related to the role of EB1 in cytoskeletal microtubule dynamics.

The interaction of FKBP52 with tubulin is mediated by a C-terminal region including the TPR domain [11]. The similarity of AIPL1 to FKBP52 is greatest in this region, despite the fact that the motifs comprising the TPR domain are degenerate. The interaction of FKBP52 with cytoplasmic dynein is mediated by the N-terminal peptidyl prolyl isomerase (PPIase) domain [5,6]. While the isomerisation activity of the PPIase domain is not required for the interaction with dynein, the structural fold of the domain is important [5,6]. AIPL1 does not exhibit a functional PPIase activity and the level of conservation of AIPL1 over this region is low [51]. Our data suggest that the ability to interact with the microtubule cytoskeleton and molecular motor machinery may not be conserved in AIPL1, although whether AIPL1 is able to bind components of the molecular motor machinery such as cytoplasmic dynein components has yet to be tested.

We next examined the co-localization of AIPL1 and the EB proteins in primary cilia in cell culture and in the structurally and functionally specialized cilia of photoreceptor cells of the human and mouse retina. Recent studies have revealed that the localization of EB1 to the base of primary cilia of cultured cells, as shown here, is necessary for microtubule minus-end anchoring at the centrosome or basal body and for effective vesicular trafficking to the cilia base, and is therefore required for the assembly of primary cilia [34,35]. AIPL1 did not co-localize with primary cilia markers, suggesting that the interaction of AIPL1 and EB1 is also not related to the function of EB1 in primary cilia assembly, but must be a specific feature of photoreceptor cell biology. In accordance with this idea, AIPL1 and the EB proteins, EB1 and EB3, localized to the connecting cilia of retinal photoreceptors. Interestingly, EB1 localized to the base of the connecting cilium in the region of the basal body and to the proximal axoneme, whereas EB3 localization was restricted to the cilia-associated centrioles and basal bodies at the base of the connecting cilium. In comparison, EB1 and EB3 both localize to the base of non-motile primary cilia in cultured mammalian cells, and EB3 additionally localizes to the distal tip of some but not all motile cilia where it might promote persistent growth of microtubule axonemes [34,35]. The distinct localization of the EB proteins in the photoreceptor connecting cilium might reflect a unique photoreceptor-specific function in microtubule minus-end anchoring and vesicular trafficking at the base of the cilium or in axonemal microtubule turnover.

Like the EB proteins, AIPL1 co-localized with cilia markers in the photoreceptor connecting cilia. However, while AIPL1 was dispersed throughout the photoreceptor cells, the EB proteins were prominently expressed in the photoreceptor connecting cilia. Therefore, it is likely that only a small fraction of the total cellular pool of AIPL1 associates with the EB proteins at the photoreceptor connecting cilium. In addition to AIPL1 and the EB proteins, proteomic analysis of photoreceptor connecting cilia also detected Hsp90 and proteasome components (www.ciliaproteome.org) [44,45]. Therefore, we propose that the AIPL1-Hsp90 photoreceptor-specific heterocomplex may indeed interact dynamically or transiently with EB proteins at the connecting cilium, and thereby have an EB-targeted role in the connecting cilium. However, whether this association occurs via direct or indirect interaction with the EB proteins has yet to be shown.

In conclusion, an interaction between AIPL1 and the EB proteins has been shown by yeast two-hybrid analysis and co-immunoprecipitation. AIPL1 did not co-localize with the EB proteins or the microtubule network in cultured cells, suggesting that AIPL1 is not involved in EB-mediated microtubule dynamics or ciliogenesis. However, AIPL1 is not normally expressed in these cells as it is photoreceptor-specific. Our data also show the co-localization of the EB proteins and AIPL1 at the photoreceptor connecting cilium. This suggests an indirect or direct association of AIPL1 and the EB proteins is photoreceptor-specific, with the function of this association yet to be resolved.
